# ‘No need to worry’: an exploration of general practitioners’ reassuring strategies

**DOI:** 10.1186/1471-2296-15-133

**Published:** 2014-07-07

**Authors:** Esther Giroldi, Wemke Veldhuijzen, Carolien Leijten, Dionne Welter, Trudy van der Weijden, Jean Muris, Cees van der Vleuten

**Affiliations:** 1 Department of Family Medicine, Maastricht University, School of Public Health and Primary Care (CAPHRI), P.O. Box 616, Maastricht, The Netherlands; 2 Department of Educational Development and Research, Maastricht University, School for Health Professions Education (SHE), P.O. Box 616, Maastricht, The Netherlands

**Keywords:** Reassurance, Doctor-patient communication, General practice, Qualitative research

## Abstract

**Background:**

In view of the paucity of evidence regarding effective ways of reassuring worried patients, this study explored reassuring strategies that are considered useful by general practitioners (GPs).

**Methods:**

In a study using a qualitative observational design, we re-analysed an existing dataset of fifteen stimulated recall interviews in which GPs elaborated on their communication with patients in two videotaped consultations. Additionally we held stimulated recall interviews with twelve GPs about two consultations selected for a strong focus on reassurance.

**Results:**

To reassure patients, GPs pursued multiple goals: 1. influencing patients’ emotions by promoting trust, safety and comfort, which is considered to be reassuring in itself and supportive of patients’ acceptance of reassuring information and 2. influencing patients’ cognitions by challenging patients’ belief that their symptoms are indicative of serious disease, often followed by promoting patients’ belief that their symptoms are benign. GPs described several actions to activate mechanisms to achieve these goals.

**Conclusions:**

GPs described a wealth of reassuring strategies, which make a valuable contribution to the current literature on doctor-patient communication. This detailed description may provide practicing GPs with new tools and can inform future studies exploring the effectiveness of reassurance strategies.

## Background

Reassuring patients that their symptoms are not caused by a serious disease is one of the most common interventions in primary care and thought to be essential to good clinical practice [1-5]. There is nevertheless a paucity of research identifying effective reassuring strategies [6], despite its importance and the described difficulty of reassuring patients [7,8]. Studies measuring the effect of reassuring statements, e.g. on normal test results, have found no effect, a temporary reduction of or even an increase in anxiety [9-13]. Failure to effectively reassure patients can lead to overutilization of health care due to unwarranted consultations and treatments, leaves patients being disabled by their condition, and can have a negative impact on patients’ quality of life [10,14,15].

There is some empirical support for approaches to reassurance used in groups of patients that are known to be highly anxious, including patients with medically unexplained symptoms (MUS) and cancer survivors. These patients tend to overestimate the likelihood of medical causes of symptoms and are not reassured by straightforward reassuring statements or after receiving normal test results [12,16]. Lower anxiety levels have been associated, however, with patients who consider the complaint to be bearable and feel they understand the seriousness of symptoms [17]. It is therefore recommended that doctors explore and acknowledge patients’ concerns [17-19] and give adequate explanations about a patient’s condition [20,21] and the meaning of normal test results [11]. It is also suggested that doctors should check whether patients have understood what is explained to them by asking check-back questions and inviting patients to summarize what has been said [16]. A recent review of Pincus et al. underpinned the reassuring effect of cognitive reassurance, i.e. changing patients’ perceptions and beliefs by promoting patients’ understanding, in patients with unexplained pain [22].

Concerns about the presence of serious illness are a common phenomenon among a much wider range of patients than the groups described above [2]. Hence, doctors should be aware of communication strategies that are effective in a variety of situations where reassurance is required.

When scientific evidence is scarce, a common strategy is to explore what experts consider to be useful as a first step toward identifying what works in practice [23]. In line with Lingard’s theory on the development of expertise in communities [24], we took the perspective that doctors are members of a community that has developed professional expertise in how to reassure patients. Given that general practitioners (GPs) see a wide variety of patients and complaints and reassurance is a feature of 70% of consultations in family practice [5], we expected GPs to have developed strategies for reassuring patients in a variety of situations. GPs also often have a long-lasting relationship with their patients, so they may have experienced the long-term effects of their reassurance.

Our aim was to identify reassuring practices that are considered useful by GPs and why as a starting point in the exploration of possible effective reassurance. As reassurance is a complex intervention that can lead to different outcomes in different situations, it is important to understand the underlying mechanisms of this intervention [25]. Recognizing these mechanisms can be instrumental in determining what works, for whom, in what circumstances, and why [25]. For this reason, our study does not solely explore GPs’ reassuring actions, but also the mechanisms they belief to be activated by their actions and the goals they try to achieve. Together these three components, i.e. action, mechanism and goal, make up a strategy. Hence we explored which reassuring strategies GPs consider as useful in their daily practice.

## Methods

### General design

We used a qualitative observational design combining two sets of stimulated recall interviews [26] with GPs (dataset A and dataset B). To identify reassuring strategies that GPs consider as useful we explored the strategies they used during videotaped consultations with their own patients. Dataset A is an existing set of interviews that explored how GPs selected communicative actions during their patient encounters [27]. Since GPs try to reassure patients in 70% of consultations [5], we expected a secondary analysis of this dataset to yield first insights with relevance to our research question. Dataset B consists of interviews conducted for the study in which GPs were prompted to reflect on reassurance in order to deepen and broaden the findings of dataset A and obtain a rich dataset of reassuring strategies. We performed a thematic network analysis using principles of grounded theory, with an iterative process of data collection and analysis and a constant comparison method [28,29].

### Ethical approval and informed consent

The Medical Ethics Committee of Maastricht University Medical Centre granted approval for our study. The participating GPs and patients gave written informed consent. Verbatim transcripts of the recorded interviews were anonymized with codes.

### Selection procedures

#### Dataset A

The GPs in the existing dataset were purposively sampled in order to obtain a variety in age, gender, number of working years and practice settings. Each GP was interviewed about two videotaped consultations that were selected by the researcher to obtain a maximum variation sample with respect to the patients’ age, gender, complaint, type of consultation and GPs’ communication techniques assessed by the instrument MAAS-Global [27,30].

#### Dataset B

We contacted eighty GPs in the Southern part of the Netherlands, with at least five years of experience in general practice and who were not recently approached for other studies by our university. GPs were sent an invitational letter containing information about the study, followed by a telephone call. If the GP worked in a group practice, his/her colleagues were approached as well. We aimed to include 10–15 participants as this number was considered to be sufficient for stimulated recall interviews [31].

A researcher watched and videotaped all consultations conducted on one morning by each of the GPs. GPs were asked to rate on a scale ranging from 0 to 10 the importance of reassurance in each of the consultations. Before their consultation, patients rated their level of concern on a scale ranging from 0 to 10 [17]. For the stimulated recall interviews, the researcher used the rating on the GPs’ reassurance scale and the rating of patients’ concern to select two consultations of each GP in which the GP aimed to reassure and the patient was concerned. In case we could not find two of such consultations, we prioritized the GPs’ rating. Reassurance needed to be a goal of the GP, otherwise they could not be interviewed about how they reassured their patients.

### Interviews

For both datasets, trained interviewers conducted the interviews shortly after the consultations. The interviewers discussed the interview procedure in depth before and during the period of data collection. GPs were asked to watch two videotaped consultations and stop the tape whenever they wished to reflect upon their thoughts, intentions and actions regarding their communication (dataset A) or reassurance (dataset B). Once the video was stopped, they were prompted further to clarify these, e.g. what did you want to achieve here, why/how do you think that works? In case they did not stop the tape at a moment the researcher considered important, the researcher could do so and invited them to reflect upon their behaviour. At the end of the interviews of dataset B, GPs were asked whether, in retrospect, they would have reassured differently and if yes, they were asked why and how. Additionally we asked them whether they use reassuring strategies in their daily practice other than those used during the two discussed consultations.

### Data analysis

All the interviews were audiotaped and transcribed verbatim. We firstly analysed dataset A. Text fragments about reassurance were selected and coded using Atlas-ti software for qualitative data analysis. Actions, mechanisms and goals were extracted from the GPs’ reflections during thematic coding. In line with thematic network theory, we constructed networks to structure and visualize relationships between global themes, consisting of the goals pursued by GPs, organizing themes, consisting of mechanisms that explained how GPs’ actions promoted the goals, and basic themes, that is the actions performed by GPs [28]. The networks helped us to obtain first insights into GPs’ reassuring practices and aspects that needed to be explored further in subsequent interviews in order to acquire a better understanding of GPs’ strategies. During the thematic analysis of the interviews of dataset B, the networks were revised and expanded. The networks were eventually used to develop a schematic table presenting a framework of goals, mechanisms, and actions related to reassuring patients.

All the transcripts were analysed independently by at least two researchers with different backgrounds: health sciences (EG) or medicine (WV/CL/DW). The researchers reached consensus on the coding through discussion. The thematic networks and the schematic table were validated through in-depth discussions between the researchers.

## Results

### Data characteristics and saturation

Table 1 presents an overview of GPs’ and patients’ characteristics of dataset A [27] and B. Characteristics of individual GPs and patients are described below every quotation.

**Table 1:** Characteristics of dataset A and B

	**Dataset A**	**Dataset B**
GPs (N)	15	12
Practices (N)	12	10
Practice settings	Mixture of solo, duo, group, urban, rural	Mixture of solo, duo, group, urban, rural
GPs’ age (mean)	47.8	49.3
GPs’ sex (% male)	53,3	66.7
GPs’ years of working experience (mean)	15.7	19.2
Patients (N)	30	24
Patients’ sex (% male)	33.3	58.3
Number of complaints (range)	1 - 4	1 - 3
Patients’ age (range)	19 - 89	2 - 86
Patients’ level of concern pre-consultation (mean)	-	4.9
GPs’ rating on importance of reassurance (mean)	-	7.9

Although there was overlap in reassuring strategies between the two datasets, dataset B resulted in a large number of additional strategies. The last eight interviews revealed no new goals and the last four interviews revealed no new mechanisms. However, in each of the last two interviews one new action was identified.

### Interviews

Average interview time for both datasets was 60–90 minutes. In both datasets, GPs most often stopped the tape to reflect upon on their actual behaviours and they were less likely to spontaneously describe their thoughts and intentions. However, when prompted they gave detailed reflections upon the reasons and purposes of their actions.

During the interviews the GPs primarily discussed strategies that they used during the two consultations and were visible on the videotape. All of the identified strategies were used during discussed consultations by at least one of the GPs, including those that were described when asking GPs whether they used strategies other than those seen in the two consultations.

### Goals that GPs pursue to achieve reassurance

**Figure 1:**
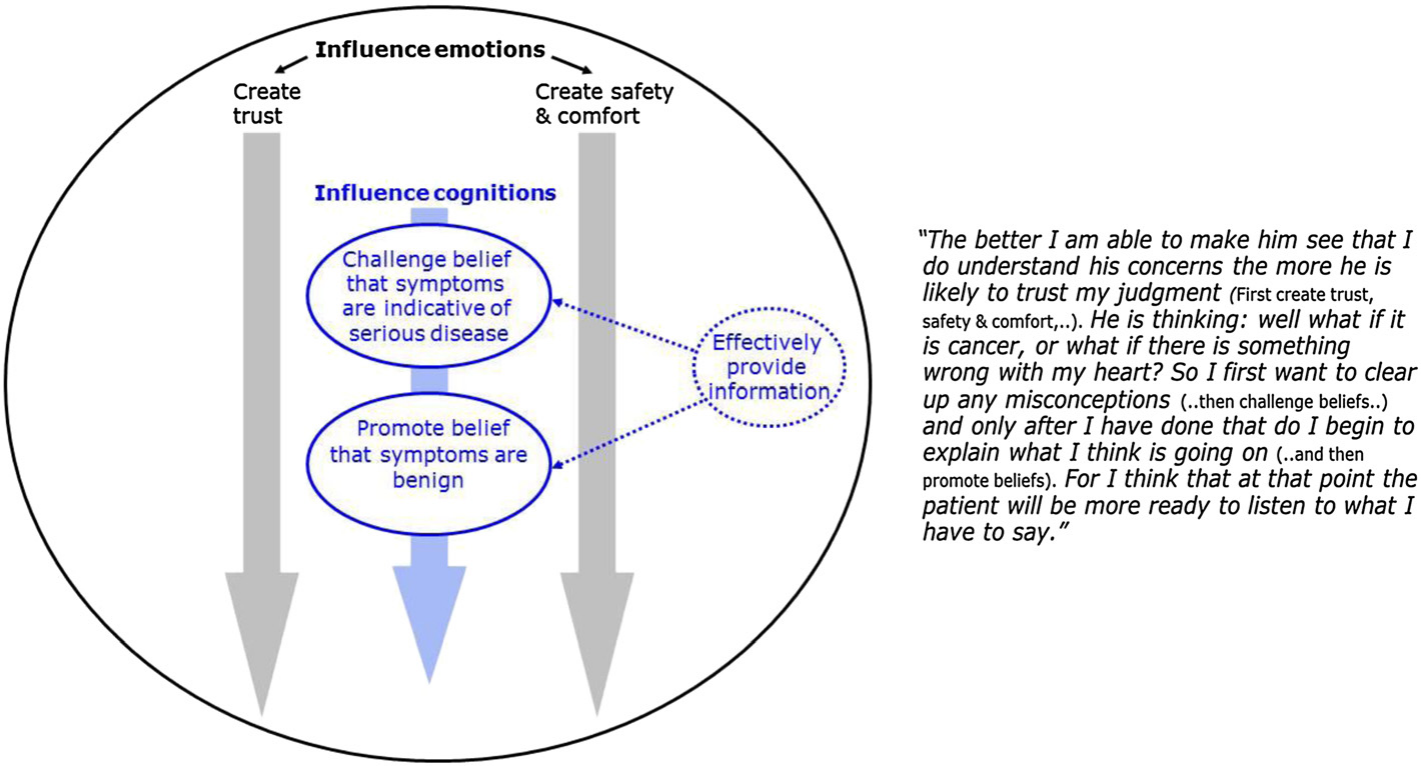
GPs’ goals in a reassuring consultation.

The goals GPs pursued to reassure patients related to the emotions and cognitions of patients (Figure 1). Emotions were involved in GPs’ actions to generate an environment characterized by trust, safety and comfort, which they considered prerequisite to effectively address patients’ cognitions. As shown in Figure 1, GPs mentioned that actions targeted at emotions could occur at different moments during the consultation, although it was often considered important to immediately start creating trust at the beginning (grey arrows). Efforts to influence patients’ cognitions were concentrated on challenging patients’ belief that their symptoms were indicative of serious disease and on promoting patients’ belief that symptoms were benign. Several GPs argued that patients’ concerns regarding serious disease should be addressed first in order to make patients more receptive to the possibility of an alternative, more harmless explanation (blue arrow). These different goals pursued within a reassuring consultation are illustrated with a quotation (Figure 1). GPs also explained that in order to successfully influence patients’ cognitions, an adequate transmission of reassuring information is essential (dotted arrows).

### Reassuring strategies: goals, mechanisms, and actions

GPs described several mechanisms that promoted the goals of influencing patients’ emotions and cognitions and several actions to activate these mechanisms. All the described strategies are listed in Table 2. Using the structure of Table 2 and illustrative quotations, we describe the goals, mechanisms and some examples of actions below. In addition, GPs described several actions to convey reassuring information to patients in an effective manner, enhancing clarity and understanding. These actions are outlined in a separate paragraph at the end of the results section.

**Table 2:** GPs’ strategies for reassurance: goals, mechanisms and actions

**Mechanisms**	**Actions**
**Goal 1. Influence emotions: create trust**	
Patient trusts GP’s expertise.	- Give detailed explanations instead of only answering the patient’s questions.
	- Show that you are fully informed about the patient’s situation.
	- Calm and unconcerned demeanour.
	- Refer to scientific evidence.
	- Emphasize experience and expertise.
Patient has trust in doctor-patient relationship.	- Inform patient honestly, also about diagnostic uncertainties.
	- Long-lasting GP-patient relationship.
	- Create comfortable atmosphere.**
	- Make patient feel heard and understood.**
**Goal 2. Influence emotions: create safety and comfort**	
Patient finds him/herself in a comfortable atmosphere.**	- Approach patient in a friendly manner.
	- Make small talk with patient.
	- Use humour.
	- Make sure the patient understands what will happen during the consultation.
	- Comfortable, homey interior of doctor’s office.
	- History taking/small talk during physical examination.
Patient feels heard and understood.**	- Allow the patient to tell his/her story.
	- Listen attentively.
	- Ask the patient to clarify statements.
	- Explore patient’s beliefs and ideas about diagnosis and treatment.
	- Pay attention to patient’s whole situation.
	- Repeat/summarize patient’s statements.
	- Announce that you are about to take the patient’s history.
	- Name patient’s concerns.
	- Acknowledge that you understand the patient’s complaint/reason for visit/concerns.
	- Respond to complaints and uncertainties expressed by the patient.
Patient feels GP takes responsibility to properly investigate, treat and monitor patient’s complaints.	- Make clear what will be done during: the consultation, history, physical examination, investigations to help diagnose the patient’s complaint.
	- Explain actions during the physical examination.
	- Perform: history focused on the feared diagnosis/careful physical examination of the part(s) of the body related to the symptoms and concerns/investigations/referral/consult with specialist/recommend specialist.
	- Repeat what was examined before discussing the findings.
	- Propose treatment that is tailored to the patient’s wishes and needs.
	- Treat symptoms that are causing anxiety.
	- Ensure continuity of care.
	- Offer opportunity for/schedule follow-up appointment.
**Goal 3. Influence cognitions: challenge belief that symptoms are indicative of serious disease**	
Patient is reassured about his/her misconceptions as GP understands patient’s beliefs and concerns.	- allow patient to tell his/her story.
	- ask open-ended/closed questions.
	- name/summarize concerns.
	- explore the burden of the complaint.
	- respond to (non-)verbal expressions of concern.
	- explore patient’s beliefs about possible causes of the complaint.
	- explore concerns early in the consultation.
	- explore concerns after sharing the findings or the diagnosis.
	- explore concerns in a setting of physical proximity (e.g. during the examination).
Patient receives information that helps him/her to conclude that the complaint is not serious, both in this consultation and when the patient may experience similar symptoms in the future.	- Emphasize reassuring signs.
	- Describe alarm signals of the feared diagnosis.
	- Ask questions related to the feared diagnosis (e.g. symptoms) which elicit answers that contradict that diagnosis.
	- Explain how the findings of history, physical examination, and other investigations rule out the feared serious diagnosis
	- Explain that if the physical examination or investigations reveal no abnormalities, the patient has the harmless condition.
	- Remind the patient of similar complaints in the past that turned out to be no cause for concern.
	- Discuss the cause of the patient’s tendency to be concerned.
Patient does not interpret abnormalities and GPs’ medical actions as indicative of serious disease.	- Play down relevance of abnormalities by explaining: the interpretation of abnormalities in test results and (ir)relevant values/that the symptoms are not necessarily related to the feared diagnosis/that the complaint should be viewed as a discomfort rather than a threat/that not normal does not necessarily imply the presence of disease.
	- Explain that history, physical examination, investigations, referral, treatment do not signify that the doctor is worried but are done: to exclude something/to reassure the patient/because it is standard procedure/because the patient experiences complaints.
Patient’s belief that there is no reason for concern is strengthened.	- state: I am not worried/I can reassure you/I am absolutely sure that serious disease has been excluded/you are worrying more than is necessary/you were thoroughly examined and no abnormalities were found.
	*-* demonstrate non-verbally that you are not worried*.*
**Goal 4. Influence cognitions: promote belief that symptoms are benign**	
Patient’s attention is shifted toward an alternative explanation.	- Explore patient’s thoughts about the harmless diagnosis.
	- Announce that you will examine the area related to harmless diagnosis.
Patient receives information that supports or suggests a harmless diagnosis.	- Correct misconceptions about a harmless diagnosis.
	- Explain what causes the symptoms.
	*-* Demonstrate the cause of the symptoms.
	- Outline a normal, realistic prognosis.
	- Explain that it is normal to experience these symptoms given the patient’s situation.
	- Make sure that the harmless diagnosis fits with the patient’s self-image.
	- Explain that the complaint is self-limiting/easy to manage.
	- Recommend home remedies.
Patient is able to talk him/herself into a reassuring conclusion.	- Let the patient tell his/her story.
	- Check whether the patient is reassured.
	- Ask questions about the harmless diagnosis which elicit answers that support that diagnosis.
Patient does not develop new worries that might overshadow the reassuring conclusion.	- Change the subject shortly after the patient has arrived at a reassuring conclusion.
	- Ignore expressions of new complaints, uncertainties, and assumptions.
	- Link newly expressed symptoms to the benign diagnosis.
	- Emphasize/show with impatience that consensus has been reached about the diagnosis.
	- Show that you are certain about the diagnosis and do not share with the patient any doubts you might have.
	- Do not perform a physical examination or make a referral.

Strategy = combination of an action, mechanism and goal.

**‘Create comfortable atmosphere’ and ‘make patient feel heard and understood’ are actions to create trust in the doctor-patient relationship. However they also contribute directly to the goal of safety and comfort as two separate mechanisms.

### Goal 1. Influence emotions: create trust

GPs believed that for patients to buy into reassuring information they needed to have faith in their GP’s expertise and in the trustworthiness of the doctor-patient relationship. They emphasized that in gaining the patient’s trust it was important to give non-verbal signs that they were confident of their judgment, because patients are likely to pick up any signal that the doctor is worried too. GPs also tried to create trust by thorough explanations, e.g. about the GPs’ procedures, the absence of serious disease and the cause of the symptoms. They thought this generated more trust in the GP’s expertise than merely answering the patient’s questions.

If someone only answers your questions it is hard to be sure what they are getting at. When someone tells you what they are doing, how things work, you give the impression: I know what I am talking about and that inspires a sort of trust, and that is reassuring.

GP 10B (GP 10 from dataset B), ♀ (female), 57 y (57 years of age) 20 exp (20 years of working experience). Patient 1, ♀, 48 y, excessive sweating (complaint for which reassurance is given at this moment)

Some GPs emphasized that trust depended on factors like a long-standing trusting doctor-patient relationship, which could not be established in one single consultation.

What helps is when you know people well. When you have seen them before, the seeds you have sown earlier, which gives them confidence that what you say is correct and that you would be honest about any doubts you might have.

GP 11B, ♀, 47 y, 17 exp. Patient 2, ♀, 47 y, hip complaints

### Goal 2. Influence emotions: create safety and comfort

GPs argued that in order to make patients feel safe and comfortable it was important to create a calm and relaxed atmosphere. This could be achieved by approaching the patient in a friendly manner engaging in small talk and using humour and by carefully explaining what would happen during the physical examination, especially when the patient was very anxious or the examination would involve actions that were not expected by the patient.

I have known him for quite some time and he is very friendly toward other people, so I try to be friendly too, to establish rapport with him so that he feels at ease and consequently is more inclined to believe and value what I say.

GP 6B, ♂, 48 y, 19 exp. Patient 1, ♂, 68 y, sudden shaking attack

GPs explained that it was important for patients to feel heard and understood by their GP.

I confirm that I can imagine that this is a burden. When people feel they are not heard, that they are talking at a wall, that you brush aside everything they bring up, they will only get more worried.

GP 11B, ♀, 47 y, 17 exp. Patient 1, ♀, 36 y, weight gain

To ensure that patients knew that their whole situation was taken into account, GPs asked about the patient’s social situation or mentioned that they understood that symptoms were worse when the patient was at home.

According to GPs, patients needed to be shown that the GP took responsibility for their complaints. This could be done by announcing and explaining the different stages of the consultation, showing that they were aware of the patient’s concerns and took them seriously.

His complaint is that he is worried. So I think if I can show him that I ask questions about his concerns he will know that his problem has my full attention.

GP 12A, ♀, 47 y, 16 exp. Patient 1, ♂, 49 y, dyspnoea

### Goal 3. Influence cognitions: challenge patients’ belief that symptoms are indicative of serious disease

GPs described that for reassurance to be effective it was not enough to simply state that the symptoms were no cause for concern, and they used a range of strategies to persuade patients that they were mistaken in thinking they were suffering from a serious disease.

#### Patient is reassured about his/her misconceptions as GP understands patients’ concerns

GPs mentioned that they aimed to understand patients’ concerns, and in dealing with patients’ cognitions they let themselves be guided by specific misconceptions expressed by the patient. That is why most GPs preferred to explore the patient’s concerns at an early stage in the consultation.

I am thinking: are you worried that it is associated with cancer? I first want to know her line of thinking. Also, discharge and lung cancer, those are totally unrelated. When I focus too much on exploring things that go in another direction without asking her what she is thinking I will be on a totally different track. In my opinion it has nothing to do with that, but to her there may be a connection and I had better go along with that.

GP 11A, ♀, 40 y, 8 exp. Patient 2, ♀, 69 y, vaginal discharge

Some GPs thought it was also effective to explore patient concerns during physical examination, as proximity creates a favourable environment for patients to share information, including concerns.

#### Patient receives information that helps him/her to conclude that the complaint is not serious

GPs described that giving patients information that negated their concerns about the presence of a serious condition could help to reassure patients during the consultation but also for the future when the patient might experience similar symptoms. GPs frequently mentioned that they described to patients the symptoms of a serious diagnosis while at the same time pointing out that these symptoms were absent.

Some GPs encouraged patients to reassure themselves. This involved asking the patient if certain alarming symptoms of the feared diagnosis were present thereby leaving it to the patient to discover that these symptoms were absent.

It is preferable that the patient says that it [the worrying symptom] is not present than that I point it out. This is clearly more reassuring. If the patient identifies the absence of worrisome symptoms that is more effective than when the GP says it is this or that.

GP 7B, ♂, 48 yrs, 14 yrs exp. Patient 1, ♂, 74 y, chest pain

Several GPs thought this strategy, as well as other strategies for helping patients to reassure themselves - which will be described later -, was more effective than telling patients their symptoms were no cause for concern, especially when patients were prone to anxiety.

GPs said that after excluding a serious disease, they wanted to make anxious patients aware of their tendency to worry, for example by explaining that it was their anxious personality or the presence of illness in the family that made them worry every time they experienced bodily sensations that seemed slightly unusual. Again, some GPs described that by asking questions, such as whether the patient experienced these complaints before a certain stressful event, they could help patients to identify what triggered their concerns and this could offer effective reassurance.

#### Patient does not interpret abnormalities and GPs’ medical actions as indicative of serious disease

When GPs informed patients about abnormalities which they thought were no cause for alarm they tried to soften the impact of the message by explaining that they described all abnormalities regardless of their clinical relevance or by explaining that deviations from normal findings had to be very large to be clinically relevant. Certain medical actions, such as referrals, were regarded as a sign of taking responsibility for the patient’s problem but could be interpreted by the patient as signifying that the GP was worried too. In such cases GPs emphasized to the patient that they took those actions to reassure the patient, not themselves .

I try to say: o.k. I will refer you to the internist but that does not mean that I think you have cancer or something. If I thought, well this looks worrying, I would make a phone call right now and you could be seen by the specialist tomorrow. But I do not share your concerns.

GP 4A, ♂, 59 y, 27 exp. Patient 2, ♀, 67 y, stool problems

#### Patient’s belief that there is no reason for concern is strengthened

Several GPs tried to persuade patients that their symptoms were not severe by explicitly stating that they saw no reason for concern. They underpinned this by showing or stating that they were not worried or by emphasizing that they had conducted a thorough examination and found no abnormalities.

No, I say there is no reason whatsoever for you to be concerned. So we have done more investigations. We have done a pelvic x-ray, a sonogram, blood tests and the outcomes so far indicate that nothing is seriously wrong, there is nothing for you to be worried about.

GP 2B, ♂, 59 y, 34 exp. Patient 1, ♀, 65 y, abdominal pain

### Goal 4. Influence cognitions: promote belief that symptoms are benign

In case GPs had insights into the actual cause of the patients’ symptoms, they mentioned that they tried to replace patients’ existing notion that their symptoms indicated serious disease with new cognitions that supported belief in an alternative explanation.

#### Patients’ attention is shifted toward an alternative explanation

GPs mentioned undertaking several actions to stimulate patients to consider less serious explanations. By exploring the patient’s thoughts about the probability of a harmless diagnosis, GPs tried to shift the patient’s attention toward that diagnosis.

He went into it himself, the stress, then I thought, yes now we have a cue to address the stress. Then I thought, I really want to hear it from him, to what extent does he feel it has something to do with it.

GP 12A, ♀, 47 y, 16 exp. Patient 1, ♂, 49 y, dyspnoea

#### Patient receives information that supports a harmless diagnosis

GPs provided information that supported an alternative diagnosis by explaining to patients what caused their symptoms, sometimes adding that these symptoms were not unusual given the patient’s situation.

She is probably menopausal and at that time many ladies notice changes in their skin, their muscles, their joints, which cause stiffness. And when you explain that, it can also be very reassuring.

GP 9B, ♂, 64 y, 38 exp. Patient 1, ♀, 49 y, joint pain

GPs described the natural course of the harmless condition to challenge patients’ prognostic misconceptions. They also tried to frame complaints in accordance with the patient’s self-image, for instance by attribution to normal ageing when the patient regarded himself as elderly. Again, this could provide reassurance not only in the present situation but also for future complaints.

#### Patient is able to talk him/herself into a reassuring conclusion

Some GPs used a strategy aimed at getting patients to reassure themselves by encouraging patients to tell their story following their own line of reasoning and asking them questions to elicit answers that would confirm a harmless diagnosis. In this way GPs stimulated patients to talk themselves into concluding that nothing was seriously wrong with them.

This is a typical phenomenon in MUS patients, when you let them talk and arrive at a conclusion, they quite often give substantial reassurance to themselves. When you as the doctor take on the role of reassurer, they will oppose anything that may be only slightly inconsistent with your story.

GP 1B, ♀, 36 y, 5 exp. Patient 1, ♀, 89 y, chest pain

#### Patient does not develop new worries that might overshadow the reassuring conclusion

It was mentioned that it was important to steer clear of opportunities for patients to develop new concerns once the GP had convinced them of the non-serious diagnosis. When patients came up with new symptoms, some GPs responded briefly to make the patient feel heard, pointing out that these symptoms were consistent with the benign explanation. By contrast, GPs sometimes deliberately refrained from responding to show these symptoms were nothing to worry about. GPs believed this was only possible in case they had already invested sufficiently in making the patient feel heard and understood in the beginning of the consultation.

GPs were aware that their actions or statements could give rise to fresh concerns, especially when patients were very anxious or were confronted with illness in the family. To prevent anxiety in these patients, GPs refrained from sharing any doubts they might have about the diagnosis or from diagnostic procedures.

This is a very insecure person, which is understandable given his history, with a mother who had a brain tumor. On my part I really think it would be useless to refer him. I will send him home worrying if I show any doubt about that. He will be stressed, thinks he has a brain tumor. You can say with 90 per cent certainty that it is a migraine. The percentage is probably even higher. Then you just have to act as if you are one 100 percent certain.

GP 5A, ♀, 34 y, 4 exp. Patient 2, ♂, 52 y, headache

This strategy contrasted with the strategy of sharing diagnostic uncertainties with patients to inspire trust in the GP’s openness or initiating investigations and referrals to show that complaints were thoroughly investigated.

### Effectively provide reassuring information

Providing information to patients was one of the cornerstones of GPs’ attempts to influence patients’ cognitions. GPs took a range of actions to ensure that patients understood and interpreted information correctly, such as repeating reassuring information and checking whether patients had understood everything. To ensure clarity, GPs used simple language without medical jargon and visual supports such as anatomical models and graphs. They also handed out information leaflets which patients could read at home to find confirmation that their symptoms are nothing to worry about. Several GPs demonstrated where the symptoms originated and explained their findings during physical examination. Most of these GPs repeated the findings when the patient was seated again after the examination. Some GPs did not share findings until after the examination because they believed that by then patients were better able to take in information.

## Discussion

The analysis of the interviews showed that GPs used a range of different strategies they considered useful when reassuring their patients. GPs specifically stressed that for effective reassurance it was important to target patients’ emotions and cognitions. Influencing emotions by promoting trust, safety and comfort was considered essential to create an environment in which cognitions could be altered successfully. By influencing cognitions, GPs aimed to challenge patients’ belief that their symptoms were serious, preferably before they tried to convince patients that their symptoms were benign. In order to successfully alter cognitions, GPs deemed it necessary to take specific actions to communicate information effectively. Depending on the situation, GPs used different actions to activate mechanisms that contributed to achieving their goals. For patients who were prone to anxiety it was considered wise to give them an active role in constructing reassuring information. For less anxious patients, GPs considered it to be sufficient to give the reassuring information.

The sequence of influencing cognitions that several GPs described was also suggested by Starcević as being useful in reassuring hypochondriacal patients [32]. Some of the actions we identified were reported in the literature to have an effect on anxiety in specific subgroups of anxious patients. In MUS patients it was shown to be effective to explain the actual cause of their symptoms, which is often psychosocial, and use information leaflets to transmit this information [21]. Re-attributing somatic complaints to a psychosocial cause can be supported by explanation and demonstration, such as explaining how symptoms can be caused by stress or demonstrating that complaints are caused by muscle tension [33,34]. Also in cancer survivors, anxiety levels decrease after adequate transmission of information, preferably using written support [20]. In both sub-groups it has been shown that feeling heard and understood by exploring and acknowledging concerns, beliefs, and ideas; showing empathy and summarizing can ameliorate anxiety [19,21,33-35].

Actions ensuring that the patient feels listened to and understands the information are included in well-known models of consultations skills such as the Calgary-Cambridge guides and the SEGUE Framework [36,37]. Our results support the notion that reassurance includes both affective and cognitive components [7]. Pincus et al. concluded that cognitive reassurance directly effects patient outcomes, while the impact of affective reassurance remains unclear [22]. The current study suggests that cognitive reassurance cannot be given effectively without affective reassurance. GPs seem to argue that affective reassurance works via an indirect pathway, i.e. by facilitating cognitive reassurance. This is in line with Epstein et al. who recommended that empathy should be expressed in the beginning of the consultation, as it facilitates biomedical inquiry, reassurance and action [15]. However, affective reassurance seems to directly reassure patients who worry about the impact of their complaints on their daily functioning and do not feel heard and taken seriously by others [38].

The majority of the strategies involved the giving of information to influence patients’ cognitions but GPs also used strategies in which they asked the patient questions to encourage them to actively construct the reassuring information for themselves. This type of strategy was believed to be especially effective in patients who were prone to anxiety. This is in line with the principle of Cognitive Behavioural Therapy to invite patients to correct their cognitive errors [39].

Importantly, several contrasting reassuring strategies were identified. For example, GPs estimated that some patients could not handle GPs’ diagnostic uncertainties or diagnostic procedures, whilst other patients needed these in order to trust the GP or to feel taken seriously. Goals such as creating trust required more attention in some consultations than in others. Some strategies that are generally believed to be counterproductive, such as not responding to new symptoms and concerns, were believed to be effective under certain conditions. Thus, GPs seemed to tailor their reassurance to the context of that specific consultation, which explains why they described such a wide variety of strategies. The context-specific and goal-directed character of doctor-patient communication has also been shown in previous research [27,40,41]. The situation-specificity of reassurance has also been demonstrated in our study exploring the patients’ perspective on reassurance, which showed that what is experienced as reassuring by patients largely depends on their specific worrying cognitions [38].

In this study we used GPs’ own recent consultations as cues for stimulated recall stimulating GPs to reflect on what really happened during their patient encounters, thereby reducing the chance of incomplete, unreliable, or unfeasible answers. However, when describing the reasons for their actions, GPs may have given post-hoc rationalizations for their behaviour.

Secondly, our findings do not allow us to make conclusions on the actual effectiveness of the described strategies. Nevertheless, the descriptions of reassuring strategies, comprising actions, mechanisms, and goals, do facilitate the formulation of specific hypotheses to be tested in systematic, experimental studies in larger populations.

Furthermore, all interviews were independently coded by researchers with an insider perspective (GP and/or medical student) and an outsider perspective (health sciences) to enhance the validity of the results. The study sample of 27 interviews is relatively large considering that 10–15 interviews is the standard for stimulated recall interviews [31], though only twelve interviews focused solely on reassurance. Saturation of data collection was achieved for goals and mechanisms but not for actions. To cover all types of actions in interviews probably requires a vast number of interviews as every context generates slightly different actions. As these additional actions are likely of rare occurrence the benefit of additional interviews seems limited. Hence, we concluded that sufficient data were available to answer our research question.

Although we believe that our results can also be of use to doctors working in clinical settings other than primary care, future studies exploring reassuring practices used by other specialties or health professionals could yield additional strategies more suitable to these settings. Emergency doctors are for instance frequently faced with extremely worried patients but have limited opportunities to build a trusting doctor-patient relationship, and may consequently rely on different strategies for reassurance.

## Conclusions

This study identified a rich variety of strategies that GPs use to reassure their patients. The descriptive framework of strategies, covering actions that activate mechanisms to achieve goals, can be helpful in answering questions about ‘what works, when, for whom and why’. Until evidence is gathered on the actual effects of reassuring strategies, this framework may provide GPs with some new tools. Thus, it would be mistaken to conclude that the strategies described in this study must be used in every patient encounter or that the structure we describe is a reflection of how every consultation should be conducted. We do believe, however, that the description may support GPs in selecting actions and mechanisms to promote reassurance that are appropriate for the situation at hand. Medical schools could adopt this approach to train students in ‘breaking good news’.

## Competing interests

The authors declare that they have no competing interests.

## Authors’ contribution

EG was involved in the design of the study, in the collection, analysis and interpretation of the data and drafted the manuscript. WV was involved in the design of the study and in the analysis and interpretation of the data and critically revised the manuscript. CL and DW contributed to the data collection and analysis and were involved in drafting the manuscript. JM, TvdW and CvdV contributed to the design of the study and critically revised the manuscript. All authors read and approved the final manuscript.

## Pre-publication history

The pre-publication history for this paper can be accessed here:

http://www.biomedcentral.com/1471-2296/15/133/prepub
